# Accidental Infection of Laboratory Worker with Vaccinia

**DOI:** 10.3201/eid0906.020732

**Published:** 2003-06

**Authors:** Nissin Moussatché, Mari Tuyama, Sayuri E.M. Kato, Ana Paula V. Castro, Brian Njaine, Regina H. Peralta, José, M. Peralta, Clarissa R.A. Damaso, Paulo F. Barroso

**Affiliations:** * Instituto de Biofísica Carlos Chagas Filho, Universidade Federal do Rio de Janeiro, Rio de Janeiro Brazil; † Hospital Universitário Clementino Fraga Filho, Universidade Federal do Rio de Janeiro, Rio de Janeiro, Brazil; ‡ Instituto de Microbiologia Prof. Paulo de Góes, Universidade Federal do Rio de Janeiro, Rio de Janeiro, Brazil

**Keywords:** Poxvirus, vaccinia virus, accidental infection, smallpox vaccination, dispatch

## Abstract

We report the accidental needlestick inoculation of a laboratory worker with vaccinia virus. Although the patient had previously been vaccinated against smallpox, severe lesions appeared on the fingers. Western blot and polymerase chain reaction–restriction fragment length polymorphism were used to analyze the virus recovered from the lesions. The vaccinia virus–specific immunoglobulin G levels were measured by enzyme-linked immunosorbent assay. Our study supports the need for vaccination for laboratory workers that routinely handle orthopoxvirus.

The smallpox vaccine, formulated with vaccinia virus, is a highly effective immunizing agent. In 1980, the World Health Organization certified that the world was free of naturally occurring smallpox, and smallpox immunization programs were subsequently discontinued (*1*). Vaccination is still recommended for particular groups, namely, healthcare workers who handle materials potentially infected with vaccinia virus or other orthopoxviruses that infect humans (*2*).

The use of vaccinia virus in laboratories is likely to increase as a consequence of international concerns about the potential use of variola (smallpox) virus as a bioterrorism weapon. The vaccine is considered safe but can produce mild to moderate disease in vaccinees and can be disseminated to their close contacts (*1*,*3*,*4*). Accidental infections have also been reported. In 1991, an accidental infection with recombinant vaccinia virus was described after a needlestick injury on the left thumb of a laboratory worker (*5*). A case of vaccinia keratouveitis has been reported after accidental ocular autoinoculation from a recent vaccination site (*6*). We now report the accidental infection of a laboratory worker who manipulated vaccinia virus–infected cells.

## Case Report

**Figure 1:**
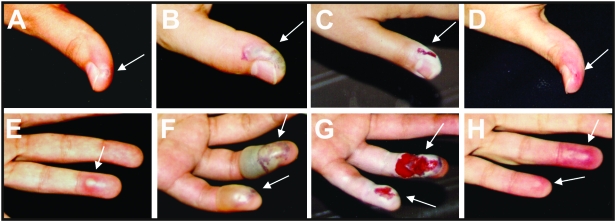
Progression of the local reaction on the left hand after accidental needlestick inoculation with vaccinia virus: thumb (A, day 4; B, day 11; C, day 12; D, day 20; fourth and fifth fingers (E, day 7, F, day 11; G, day 12; H, day 20). Lesions were surgically excised to remove necrotic tissue on day 11. Arrows indicate the lesion areas.

A 26-year-old healthy laboratory worker, previously vaccinated against smallpox in childhood, sought treatment in March 2002 with a history of pain followed by the appearance of erythema and a pustule on the left thumb (Figure 1A). These symptoms appeared 3 days after she experienced an accidental needlestick while working with material from a vaccinia virus (strain WR)–infected cell culture during a virus purification procedure. Local symptoms worsened, and on the days 5 and 6, respectively, she noticed new pustules on the fourth and fifth fingers of the same hand (Figure 1E). Axillary lymphadenopathy was noticed on the day 6 after the accident. On day 8, necrotic areas around the lesions and a large erythemathous lesion appeared on the left forearm. On day 9 after inoculation, the local lesions worsened and amoxicillin/clavunate (1,750/250 mg per day) was administered because of a clinical suspicion of secondary bacterial infection (Figure 1B, F). The hand lesions were surgically excised to remove the necrotic tissue, and pustular fluid was collected for analysis (Figure 1C, G). After the surgical procedure, the patient improved slowly until she made a full recovery (Figure 1D, H), and the lesions healed in approximately 3 weeks.

## Results

**Figure 2:**
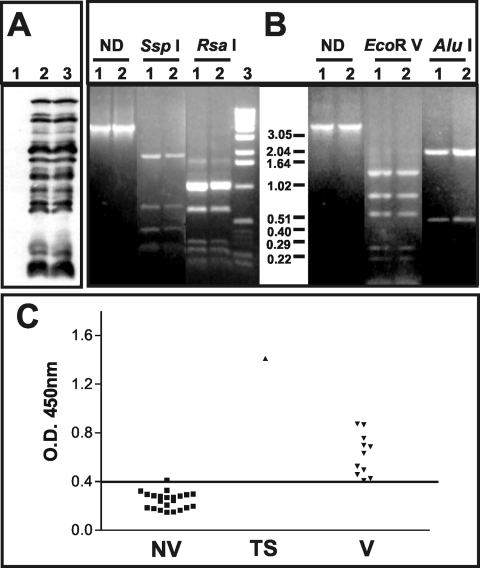
Characterization of the pustular fluid and serologic responses to vaccinia virus antigens. A) Western blot analysis of BSC-40 cells mock-infected (1); infected with vaccinia virus WR (2); or infected with 20 μL of the pustular fluid (3). Molecular weights are expressed in kDa. B) Polymerase chain reaction–restriction fragment length polymorphism analysis of vaccinia virus genome regions. Amplicons corresponding to the A24R gene or the segment between the B9R and B14R genes were digested or not (ND) with the restriction enzymes indicated on the top of the figure. (1) vaccinia virus–WR; (2) clinical sample. C) Detection of vaccinia virus–specific immunoglobulin G antibodies in serum samples from nonvaccinated (NV) and vaccinated persons (V) and the test subject (TS) was performed by enzume-linked immunosorbent assay, and the results are expressed as optical density 450-nm readings. The horizontal bar indicates the cut-off for the test.

Pustular fluid from the lesions was collected and tested for the presence of bacteria and virus. The Gram stain and cultures were negative for bacteria. When a diluted sample of the pustular fluid was added to BSC-40 (monkey kidney) cell culture, a poxviruslike cytopathic effect was evident after 48 h of infection (data not shown). Vaccinia virus proteins were detected in infected cells by 12% sodium dodecylsulfate–polyacrylamide gel electrophoresis (SDS-PAGE), followed by Western Blot analysis with rabbit antiserum raised against total vaccinia virus proteins as described before (*7*). The protein profile was indistinguishable from that of the WR strain of vaccinia virus currently used in the laboratory (Figure 2A). The presence of vaccinia virus genome in the pustular fluid could be demonstrated by polymerase chain reaction (PCR), followed by restriction fragment length polymorphism (RFLP) of the phenol-chloroform–extracted DNA from BSC-40 cells infected with the clinical sample for 24 h at 37°C (*8*). Total DNA isolated from cells infected with the vaccinia virus–WR was used as reference. Two regions of the vaccinia virus genome were analyzed by using the following PCR primers: A24Rfwd 5′ATGAAAAAAAACACTGATTC and A24Rrev 5′TTACACCAGAAAAGACGGCT; B9Rfwd 5′GACTAAATATTCATAA and B14Rrev 5′TACTAAAGTTCCGTCATC. The A24R gene was used as marker for the nonvariable region of the virus genome, and the PCR amplicons were digested with the endonucleases *Ssp*I and *Rsa*I (New England Biolabs, Beverly, MA, USA), as recommended by the manufacturer. The variable region of vaccinia virus genome was investigated by amplifying the DNA segment from the B9R to B14R genes and digestion of the amplicons with *Eco*R V and *Alu*I (Life Technologies, Rockville, MD, USA), as recommended. The digestion products were analyzed by using 1.2% agarose gels. The restriction patterns obtained for both regions in the test sample were identical to the profiles observed with the genome of vaccinia virus–WR (Figure 2B).

Serum collected from the patient day 20 after the initial inoculation was tested for vaccinia virus–specific immunoglobulin (Ig) G response by enzyme-linked immunosorbent assay (ELISA) as described (*9*,*10*). Purified vaccinia virus (1 μg/mL in 0.05 M carbonate buffer, pH 9.6) was used as the antigen, and the serum samples were diluted 1/100. Bound antibodies were detected with peroxidase-labeled, anti-human IgG (Biolab Diagnóstica, São Paulo, Brazil) dulated 1/8,000 as described (*9*,*10*). The optical density (OD) values were obtained with a microtiter plate spectrophotometer at 450 nm (BioRad, Model 3550 UV, Bio-Rad Laboratories, Hercules, CA, USA). The test serum specimen was compared to a panel of serum specimens from 22 unvaccinated persons and 11 persons who had been vaccinated some time previously, including a sample from the laboratory worker taken 6 years before the accident. When we compared the serum specimens collected before and after the accident, we observed an increase by a factor of 3.5 in the IgG-antibody response to vaccinia virus (Figure 2C). Furthermore, the vaccina virus–specific IgG levels in the test serum were 1.6 to 2.8 times higher than the levels in the panel of positive control samples and >5 times higher than levels in naive persons. Together, these results confirm that after the recent accident, a productive infection was found in the lesion and an immune response to vaccina virus was elicited.

## Conclusions

Accidental infection with live pathogens by healthcare and laboratory workers has been frequently reported (*11*,*12*). The risk of infection cannot be avoided, although it can be prevented or minimized by safety measures. In some cases, vaccination of the workers is the best way to prevent the disease; however, vaccines are not always available.

We report the response of a laboratory worker to an accidental needlestick inoculation with vaccinia virus in 2002. After the accident, typical symptoms of vaccinia infection developed in the worker, followed by full recovery 4 weeks later. Vaccinia virus could be reisolated from the pustular fluid, and no major variation from the original seed virus was detected. Although the patient had been vaccinated against smallpox >20 years ago, a serum sample isolated 6 years before the accident showed a level of vaccina virus–specific IgG antibodies approximately 2 times higher than the level in naive persons. This level of humoral immunity was not able to prevent the progression of the infection as would be expected if she had been vaccinated recently. This result indicates that despite the high IgG levels induced after vaccina virus inoculation, persons vaccinated for >20 years are no longer fully protected against vaccina virus infection and could be vulnerable to variola virus or other orthopoxviruses that infect humans.

Nevertheless, we should consider some aspects of this accident that are not common in other situations (e.g., revaccination). The amount of virus in the needle before the accident was approximately 1,000 times higher than the amount in the vaccine preparations used for smallpox vaccination (*1*). Even in a recently vaccinated person, a response to an infection of such high magnitude will most likely result in a local lesion. However, the question of whether a major reaction with severe symptoms would emerge in this hypothetical situation remains. Usually, a severe reaction has occurred only when a long period has elapsed after vaccination (*1*). Therefore, after a properly conducted risk assessment, laboratory workers vaccination should be considered as an occupational protection measure against accidental exposure to orthopoxviruses. The results of this study support the current Advisory Committee on Immunization Practices guidelines that recommend a 1-year vaccination regimen for workers who handle low-virulence poxvirus and a 3-year regimen for workers that handle high-virulence strains.
